# Improving the Acceptability and Implementation of Information and Communication Technology–Based Health Care Platforms for Older People With Dementia or Parkinson Disease: Qualitative Study Results of Key Stakeholders

**DOI:** 10.2196/58501

**Published:** 2024-06-27

**Authors:** Mona Ahmed, Mayca Marín, Pilar Gangas, Ellen Bentlage, Claudia Louro, Michael Brach

**Affiliations:** 1 Institute of Sport and Exercise Sciences University of Münster Münster Germany; 2 Institute of General Practice and Family Medicine University Hospital Bonn, Venusberg-Campus Bonn Germany; 3 Association Parkinson Madrid Madrid Spain; 4 International Foundation for Integrated Care Oxford United Kingdom; 5 Kinetikos Health Lisbon Portugal

**Keywords:** acceptability, implementation, neurodegenerative diseases, Parkinson disease, dementia, chronic diseases, health care technologies, older people, stakeholders, information and communication technology, ICT, user-centered design, co-design

## Abstract

**Background:**

The management of neurodegenerative diseases (NDDs) in older populations is usually demanding and involves care provision by various health care services, resulting in a greater burden on health care systems in terms of costs and resources. The convergence of various health services within integrated health care models, which are enabled and adopted jointly with information and communication technologies (ICTs), has been identified as an effective alternative health care solution. However, its widespread implementation faces formidable challenges. Both the development and implementation of integrated ICTs are linked to the collaboration and acceptance of different groups of stakeholders beyond patients and health care professionals, with reported discrepancies in the needs and preferences among these groups.

**Objective:**

Complementing a previous publication, which reported on the needs and requirements of end users in the development of the European Union–funded project PROCare4Life (Personalized Integrated Care Promoting Quality of Life for Older People), this paper aimed to report on the opinions of other key stakeholders from various fields, including academia, media, market, and decision making, for improving the acceptability and implementation of an integrated ICT-based health care platform supporting the management of NDDs.

**Methods:**

The study included 30 individual semistructured interviews that took place between June and August 2020 in 5 European countries (Germany, Italy, Portugal, Romania, and Spain). Interviews were mostly conducted online, except in cases where participants requested to be interviewed in person. In these cases, COVID-19 PROCare4Life safety procedures were applied.

**Results:**

This study identified 2 themes and 5 subthemes. User engagement, providing training and education, and the role played by the media were identified as strategic measures to ensure the acceptability of ICT-based health care platforms. Sustainable funding and cooperation with authorities were foreseen as additional points to be considered in the implementation process.

**Conclusions:**

The importance of the user-centered design approach in ensuring the involvement of users in the development of ICT-based platforms has been highlighted. The most common challenges that hinder the acceptability and implementation of ICT-based health care platforms can be addressed by creating synergies among the efforts of users, academic stakeholders, developers, policy makers, and decision makers. To support future projects in developing ICT-based health care platforms, this study outlined the following recommendations that can be integrated when conducting research on users’ needs: (1) properly identify the particular challenges faced by future user groups without neglecting their social and clinical contexts; (2) iteratively assess the digital skills of future users and their acceptance of the proposed platform; (3) align the functionalities of the ICT platform with the real needs of future users; and (4) involve key stakeholders to guide the reflection on how to implement the platform in the future.

**International Registered Report Identifier (IRRID):**

RR2-10.2196/22463

## Introduction

As the global population is aging, the prevalence of neurodegenerative diseases (NDDs), such as dementia and Parkinson disease (PD), has increased. In Europe, the overall prevalence of dementia in people aged between 60 and 64 years is only 0.6% on average, increasing to 40.8% among those aged 90 years or older. It is estimated that the incidence of PD has doubled over the last 25 years, increasing faster than any other neurological disorder. Both dementia and PD are of a progressive nature, and are among the major reasons for disability and dependency among older people [1-3]. Furthermore, the management of NDDs is usually demanding and involves various health care services, resulting in a greater burden on the health care system in terms of both costs and resources [4-8]. The convergence of various health services within integrated health care models, which are enabled and adopted jointly with information and communication technologies (ICTs), has been identified as an effective alternative health care solution [9-13].

Previous literature has highlighted the potential of ICTs offered in a wide variety of means for supporting the monitoring and management of health conditions [14]. Considering NDDs, ICTs have the advantages of improving patients’ quality of life and quality of care, and reducing health care costs [15,16]. However, their widespread implementation faces formidable challenges [17,18]. Some of these challenges are related to technology complexity, resistance to change, the low digital skills of intended end users, and the costs associated with deployment [19-21]. All of the aforementioned challenges are complicated due to the fact that integrated ICT health care solutions are linked to different groups of stakeholders, with reported discrepancies in their needs and preferences [22-24].

Alongside patients, caregivers, and health care professionals, who are referred to as end users, the implementation of ICT interventions involves other key stakeholders, such as policy makers, technology designers, and health care managers. In fact, supporting the successful implementation of technology for older people requires extensive work on various levels, including political, organizational, managerial, and scientific levels. This, in turn, facilitates the required changes with respect to health care and process provision [18,25]. Furthermore, engaging other stakeholders in the ICT development process helps to strengthen the likelihood that their current and future needs and requirements will be considered when advancing to real-life adoption and that the implementation process will be well coordinated, which will minimize disruption and increase the chances of aligning the process with the needs and priorities of all involved parties, thus ensuring successful adoption and sustainability [26-30]. Regardless of the identified benefits, the actual involvement of other key stakeholders in both the development and implementation of integrated ICT health care interventions remains suboptimal [17].

PROCare4Life (Personalized Integrated Care Promoting Quality of Life for Older People) is a European Union–funded project that aims to contribute to improving the quality of life and quality of care of older people living with NDDs through the development of an ICT-based health care platform to be used among patients, caregivers, and health care professionals. The user-centered design (UCD) provision of the project started with the co-design and validation process, where future end users needed to be considered from the earliest stages. The needs and requirements fieldwork conducted by the PROCare4Life multidisciplinary team went beyond the inclusion of potential end users and extended to other key stakeholders involved in the different areas of the NDD health care process [31,32].

This paper complements a previous publication [33] that reported on the needs and requirements of PROCare4Life end users for the development of an ICT-based integrated health care platform. The general approach of the study focused on identifying the aspects that PROCare4Life should consider to achieve success in its acceptance and promote its future usage, and identifying the areas to be improved across all development phases since the initial ideation design of PROCare4Life. Aiming to shed light on the opinions of other key stakeholders who took part in the study from the areas of academia, media, market, and decision making, we conducted a further analysis of the data obtained from this complementary perspective. In this paper, we report the identified results of this analysis, including participants’ opinions on improving the acceptability and implementation of an integrated ICT-based health care platform for NDDs.

## Methods

### Study Design and Setting

This study is part of a larger mixed methods research that was conducted in 2020 by the PROCare4Life consortium, which aimed to identify the needs and requirements of end users and other key stakeholders for the development of an ICT-based health care platform targeting older people with NDDs and comorbidities. The study protocol and the results primarily related to the end users have been published elsewhere [32,33]. This paper focused on the qualitative assessment that considered the opinions of key stakeholders from the areas of academia, media, market, and decision making, who were involved in health care related to NDDs.

The study included 30 individual semistructured interviews that took place between June and August 2020 in 5 European countries (Germany, Italy, Portugal, Romania, and Spain). Interviews were mostly conducted online, except in cases where participants requested to be interviewed in person. In these cases, all PROCare4Life COVID-19 distancing measures were applied [34].

### Participants

Participants representing key stakeholders were included if they did not have any conflicts of interest related to the objectives of PROCare4Life or the consortium, were aged ≥18 years, were able to provide consent, and were working or were considered experts in any of the following four areas: (1) market (potential buyers or investors of the technologies and services piloted by the project [eg, health care providers]); (2) policy and decision making (stakeholders having the power to regulate or integrate project results into their scope, be it a country, region, or organization [eg, political representatives, people implementing change in health care provision, and health-related authorities]); (3) academia (stakeholders from the scientific and cultural community, who can use the project results and outcomes in future research [eg, researchers in universities and research centers]); and (4) media (stakeholders from the media community, who can expand our reach to identified target groups and the general public [eg, press]).

All potential participants were approached by a member of the PROCare4Life team and received information about the project via email. Prior to the interviews, personalized video calls were also offered to potential participants to explain more about the study’s aims and clarify any potential doubts.

### Data Collection and Analysis

Individual interviews with open-ended questions were conducted, and each interview lasted between 30 and 45 minutes and focused on the following topics: sociodemographic data; opinions regarding ICT-based health care platforms for older people with dementia and PD, with identification of possible facilitators, barriers, and implementation prerequisites; and suggestions to be considered in designing an ICT-based health care platform, which would enhance its marketing potential and acceptability by target users.

Within the scope of an extensive evaluation, it became apparent over time that the complexity and diversity of the gathered information had significant potential, which supported revisiting the data and conducting further analysis. Collected data were recorded and transcribed verbatim according to the guidelines provided by the research team. Each interview was transcribed and revised by 2 researchers from each project site. In case the interview was conducted in a language other than English, an additional translation step was performed. The translation process involved native speakers of both the interview language and English. A deductive-inductive qualitative data analysis was conducted using MAXQDA software (version 20) [35]. The thematic analysis process [36] started with the reading of all interviews by 2 researchers independently, and both took notes and documented their observations. The initial coding was then conducted according to a framework derived from the question categories in the interviews and provided by the main researcher. Further open coding was performed inductively. Subsequently, 2 discussion sessions among the main researcher and 2 other involved researchers were held, and corresponding themes and illustrative quotes were identified and finalized.

### Ethical Considerations

The study protocol was approved by local ethics committees in Germany (University of Münster, number 020-37-MB), Italy (Casa di Cura del Policlinico; now named Casa di Cura Igea, number 493-2020), Portugal (Campus Neurológico Sénior, number 10-20), Romania (Universitatea de Medicina si Farmacie "Carol Romania Davila" din Bucureşti, number 7/10.06.2020), and Spain (Asociación Parkinson Madrid, number 20/453-E). The organizations conducting this study enacted an additional data handling agreement prior to commencing any processing of personal data, according to legal regulations and following good research practices.

Participation was entirely voluntary, and participants had the right to withdraw from the study at any time, without giving reasons or experiencing any disadvantages. Adhering to the standards of Good Clinical Practice and the International Conference on Harmonization standards, once the study was fully explained, written or digital informed consent was obtained from each participant before any study-related procedures.

## Results

### Participants

A total of 30 stakeholders completed the interviews (Table 1), including 6 media stakeholders, 8 market stakeholders, 8 decision makers, and 8 participants who worked in the academic field. Most of the participants were from Germany (11/30, 37%) and Portugal (10/30, 33%). More than half of the participants were male (16/30, 53%), and 47% (14/30) were aged between 46 and 65 years. Moreover, 40% (12/30) of the participants were PhD holders and had over 20 years of working experience.

**Table 1:** Characteristics of participants across countries and stakeholder subgroups.

Characteristic			Value (N=30), n (%)
**Type of stakeholder**			
	Media stakeholder	6 (20)	
	Market stakeholder	8 (27)	
	Decision maker	8 (27)	
	Academic stakeholder	8 (27)	
**Country**			
	Germany	11 (37)	
	Italy	5 (17)	
	Portugal	10 (33)	
	Romania	2 (7)	
	Spain	2 (7)	
**Gender**			
	Female	14 (47)	
	Male	16 (53)	
**Age (years)**			
	18-30	2 (7)	
	31-45	8 (27)	
	46-65	14 (47)	
	66-75	5 (17)	
	Not reported	1 (3)	
**Education level**			
	Postsecondary school	2 (7)	
	Bachelor’s degree	1 (3)	
	Master’s degree	11 (37)	
	PhD	12 (40)	
	Not reported	4 (13)	
**Experience (years)**			
	1-5	4 (13)	
	6-10	6 (20)	
	11-20	7 (23)	
	>20	12 (40)	
	Not reported	1 (3)	

### Evaluation Outcomes

Based on the data gathered and analyzed across the semistructured interviews, our participants were largely aligned in suggesting some ideas when approaching potential end users. Considering the possible measures for improving the acceptability and implementation of an ICT-based health care platform, we identified 2 themes and 5 subthemes.

#### Theme 1: How to Improve the Acceptability of an ICT-Based Health Care Platform

In this theme, participants shared their opinions regarding aspects for improving the acceptability of an ICT-based health care platform among end users in the NDD care process. Table 2 presents the subthemes, codes, and sample quotations corresponding to this theme.

**Table 2:** Qualitative data analysis results (subthemes, codes, and quotations) corresponding to theme 1 (how to improve the acceptability of an information and communication technology–based health care platform).

Subtheme and code			Quotation ID		Interviewee profile		Sample quotation
**Subtheme 1.1: User engagement**							
	Understand the users’ needs	Q1.1a		Market stakeholder from Italy		“All the aspects related to users' perception and experience should be taken into consideration in order to tip the perspective and generating a demand instead of an offer.”	
	User-center approach	Q1.1b		Market stakeholder from Italy		“If the user-centered methodology work, it will be relevant to receive the feedback about the platform use from different users.”	
	Early involvement of target users	Q1.1c		Academic stakeholder from Germany		“I think the system has to be developed very closely with people from the target group and always be tested from the beginning. So, any approach should be discussed and worked out with older people and then I think that this access to the different methods needs to be considered.”	
	Conduct regular usability assessments	Q1.1d		Academic stakeholder from Romania		“This requires performing quantitative and qualitative research; quantitative in which you apply some validated questionnaires on quality of life or something else, and qualitative by means of interviews, such as this, you talk to the patient, if possible, with the family/caregiver.”	
**Subtheme 1.2: Training and digital divide**							
	Digital illiteracy and resistance to technology	Q1.2a		Decision maker from Portugal		“The barriers essentially have to do with the lack of knowledge. The digital evolution is constant and requires continuous training of all people in the health area. There is some resistance from the elderly to technologies.”	
	Provide education for end users	Q1.2b		Academic stakeholder from Portugal		“All people involved in this project should have the information about the main objectives of the project and about all the devices involved regarding the data. It's important to give them some education.”	
	Methods for education	Q1.2c		Market stakeholder from Italy		“Healthcare providers can be informed through continuing education, webinars, seminars, and conferences.”	
	Methods for education	Q1.2d		Decision maker from Portugal		“Define methodology and concepts very well. Carry out pilot projects that give confidence to health professionals and disseminate results.”	
	Training	Q1.2e		Market stakeholder from Germany		“I think people need to be trained as a prerequisite.”	
	Provide evidence-based data	Q1.2f		Academic stakeholder from Germany		“To know about the acceptability and usability is super useful, whether it works or not, and to know which things work and which don´t work, so these results are super valuable for sure. I think you should avoid the issue of promoting your system, I don´t know if there is a risk of this and there could be a bias of being positive about the system, I think that can really help to be critical and have a self-criticism built in the way you report the results, and avoid trying to sell your system to the research community, and that can be a very strong point if you can minimize the risk of bias.”	
**Subtheme 1.3: Usage of the media**							
	Which media channels are preferred by the old population	Q1.3a		Media stakeholder from Spain		“If we follow reliability then our target group prefers the radio first, then newspapers, TV and internet the last. So, the perfect option for them it would be that their doctor would be talking about this kind of project in a local radio.”	
	Social media	Q1.3b		Media stakeholder from Portugal		“Social media can be an entry point, a way to capture attention and visibility. More importantly, they allow to target users according to their profile.”	
	Create your own media channel	Q1.3c		Media stakeholder from Germany		“You could also create a PROCare4Life group, so that the relatives can exchange information (…) But I think social media are a good thing in any case. But you also have to place the contributions well (….) Or you can actually create your own social media.”	

#### Subtheme 1.1: User Engagement

There was an agreement among participants that involving potential end users in the development process of ICT-based health care platforms from inception is fundamental. This involvement should start from the beginning of the project and continue in the design phase in a way that can create a need for the proposed platform rather than an offer (quotation ID Q1.1a in Table 2). Participants also pointed out that adopting the UCD approach is essential (quotation ID Q1.1b in Table 2), which can ensure that potential end users are involved from the early stages of the proposed platform design and throughout the implementation phase (quotation ID Q1.1c in Table 2). Furthermore, the processes of collecting and reassessing user experience and acceptance frequently via qualitative and quantitative studies need to be incorporated and properly planned throughout the development phase (quotation ID Q1.1d in Table 2).

#### Subtheme 1.2: Training and Digital Divide

Digital illiteracy or lack of knowledge about using technological devices among patients, caregivers, and health care professionals was reported as a barrier, and some end users tend to avoid or even reject using innovative technologies (quotation ID Q1.2a in Table 2). It is important to foster the knowledge of potential end users involved in the care process through education about the proposed platform, including its benefits, integrated devices, and usage (quotation ID Q1.2b in Table 2). Education can be provided using multimedia methods adapted to the target population. Information about the methodologies and concepts adopted in the design and development of the proposed platform might be relevant for some end users (mostly health care professionals and managers). Some of the means to provide information to a wider audience might involve conferences or dissemination activities in order to provide people with evidence that is convincing for integrating the platform into their working routine (quotation IDs Q1.2c and Q1.2d in Table 2). Furthermore, providing training opportunities on how to use technological devices is important for overcoming digital literacy barriers (quotation ID Q1.2e in Table 2). At later stages, participants in the co-design activities can receive back reflections of the early or preliminary results obtained from the pilots conducted during the development process with potential end users. This can be achieved by providing evidence-based material regarding the strengths and limitations of the current version of the proposed platform (quotation ID Q1.2f in Table 2).

#### Subtheme 1.3: Usage of the Media

Using media channels for advertising and promoting the proposed platform was discussed during the interviews. Participants highlighted the role of the media as part of the marketing strategy for an ICT-based health care platform. Elaborating on this subtheme, our participants referred to the heterogeneity of target end users when it comes to their media preferences. It was stated that older populations prefer the radio, newsletters, and television, and sometimes use internet-based media (quotation ID Q1.3a in Table 2).

On the other hand, social media was seen as an entry point that can capture visibility for target end users (quotation ID Q1.3b in Table 2). Therefore, some participants even suggested creating a media channel for each project, where real stories derived from the pilot participants could be shared, agreeing that such content is powerful and could increase acceptability among older populations and other user groups (quotation ID Q1.3c in Table 2).

#### Theme 2: Suggestions for Facilitating the Implementation of an ICT-Based Health Care Platform

In this theme, participants elaborated on measures that can be considered in the implementation phase of digital health care platforms. They suggested assuring funding sustainability and cooperation with different authorities. The identified subthemes, codes, and sample quotations are illustrated in Table 3.

**Table 3:** Qualitative data analysis results (subthemes, codes, and quotations) corresponding to theme 2 (suggestions for facilitating the implementation of an information and communication technology–based health care platform).

Subtheme and code			Quotation ID		Interviewee profile		Sample quotation
**Subtheme 2.1: Funding sustainability**							
	Funding is an initial barrier	Q2.1a		Decision maker from Portugal		“The financial issue may only be an initial barrier. But it is all relative, it depends on the service provided. If it is really good, a service of excellence, it is no longer considered an expense and becomes an investment. We must have enough data to prove it and be seen as an asset, with results. The beginnings are always hard.”	
	Health and social systems should provide funding	Q2.1b		Decision maker from Spain		“It is not an easy task, but it should be financed by both the health and social systems.”	
	Third-party funding	Q2.1c		Decision maker from Germany		“Applying for funds through projects.”	
	Provide different price offers	Q2.1d		Market stakeholder from Germany		“In my opinion the price should be practically depending on the type of digital solution, it would have to be divided somehow. I can’t demand the same price from someone who only uses one sensor as from someone who uses everything else. I think that would have to be graded according to the type of performance.”	
**Subtheme 2.2: Collaboration with authorities**							
	Collaboration between local and general authorities	Q2.2a		Decision maker from Italy		“A more efficient and constructive cooperation between local decision makers and higher, national ones would facilitate the implementation of an integrated care system. Then, an informative action inside the hospital to raise awareness in patients and health-professionals about this kind of systems would be beneficial (…) Individual national organizations first, and then the regional ones, and their regulations, should be considered in order to put in place an integrated care system like this.”	
	Political and social cooperation	Q2.2b		Decision maker from Portugal		“Political will and bringing partners to the project. Having a strong and robust social network, which is the basis for developing such a project. There is a strong desire by the municipalities to work on a model of this nature.”	
	Importance of communication between different authorities	Q2.2c		Decision maker from Spain		“Communication channels. Right now, there is no communication channel between the health and social services, but there are no efficient communication channels between the health services themselves or between the social services either.”	

#### Subtheme 2.1: Funding Sustainability

Participants referred to financing issues as an initial barrier to implementing and maintaining an ICT-based platform. However, positive results and benefits obtained through the development and implementation of the platform can contribute to overcoming this barrier (quotation ID Q2.1a in Table 3). To provide sustainable funding for digital health care solutions, participants proposed several approaches, such as finance supported by social health care systems and health insurance companies (quotation ID Q2.1b in Table 3) and project-based funds (quotation ID Q2.1c in Table 3). A software as a service funding model was also suggested, with providing different customized packages that fit individual needs and usage patterns at affordable prices (quotation ID Q2.1d in Table 3).

#### Subtheme 2.2: Collaboration With Different Authorities

Another suggestion that was repeated in the interviews was the prioritization of cooperation with different authorities. For ensuring successful implementation of digital health care solutions, participants pointed out the importance of bringing local and regional authorities together. Local organizations need to contribute to raising awareness of the expected benefits from such solutions before their implementation by regional authorities (quotation ID Q2.2a in Table 3). Interviewees also mentioned that including political and social partners together is one of the bases for developing and implementing digitally enabled integrated health care solutions (quotation ID Q2.2b in Table 3). There is a need for creating communication channels between all the different sectors involved in the health care process and digitalization of the society, with efficient collaborations between health and social care services (quotation ID Q2.2c in Table 3).

## Discussion

### Principal Findings

This study addressed strategies needed to improve the acceptability and implementation of an integrated ICT-based health care platform. Beyond potential end users (ie, patients, caregivers, and health care professionals), we brought in the voices of other stakeholders linked to health care technologies in the areas of academia, media, market, and decision making. The majority of the participants identified the importance of the UCD approach for including end users in the development process and exploring their needs. End-user engagement impacts the initial acceptability and sustained usage of ICT-based health care solutions, as reported by Nadal et al [37]. Hence, the early engagement of end users starting from obtaining their feedback regarding the design of the solution and moving to performing regular usability assessments throughout the development and implementation processes is needed.

Despite the general acceptance of ICT solutions by older populations, their families, and health care professionals [33,38,39], participants in this study stated that the lack of knowledge about the benefits and design of the technology and the complexity in using and managing the technology are common challenges for ICT adoption in NDD health care. Providing education about the design, aims, and benefits of ICT-based solutions was therefore proposed. Additionally, conducting conferences, workshops, and other dissemination activities to inform potential end users about the proposed solutions has been suggested as part of educational activities, which can help overcome these challenges and facilitate the adoption of ICT in health care [40-46]. Furthermore, providing continuous training to all potential end users is needed and should be considered from the early stage of development. In particular, this is important for digital platforms that include multiple devices. Tailored training programs should consider the various digital skills of the intended end users, with the aim of simplifying the design and thus increasing acceptance [47]. On the other hand, participants from the scientific field pointed out the importance of reflecting on the usability results obtained from target users and making these data available to the public. This finding complements other evidence indicating that the validation and assessment of digital interventions via trials involving older adults are critical and can impact acceptance and health decision making [48].

Another important finding was regarding the role of media channels. Our participants stated that media channels contribute to informing potential target groups about innovative ICT platforms, raising awareness, and fostering a positive attitude toward available ICT solutions. Similar to previous literature, these contributions support the acceptance and successful implementation of ICT solutions in health care [49,50]. While the use of social media has increased rapidly across various age groups in recent years [51], our participants highlighted the heterogeneity of preferences among older people with chronic diseases. Traditional media channels, such as radio and television, were considered the first choices for use when approaching this target group. It is also noteworthy that some of the participants recommended creating a media channel dedicated to each project, where personal narratives, success stories, and challenges faced by end users can be shared.

Similar to the findings in previous research [52,53], our participants referred to costs and sustainable funding as common barriers to implementation across digital health care domains. Addressing these barriers would require collaborative multidisciplinary work on various levels, such as cooperation between social and health systems. Some of the participants voiced the need for providing different pricing models, which are both personalized for the users’ needs and affordable. In line with what has been reported by Frishammar et al [39], providing opportunities for flexible and personalized pricing models is only possible through active collaborations between developers and policy makers in digital health care. Therefore, the majority of participants highlighted the need for bringing relevant authorities together when developing and implementing successful ICT-based health care solutions for older people. Furthermore, finding a medium for communication is critical. This is of great importance as communication among various authorities is crucial to guarantee cross-disciplinary collaboration, in which ICT solutions can be put to work effectively [17,18].

### Recommendations for Research on Users’ Needs

**Figure 1:**
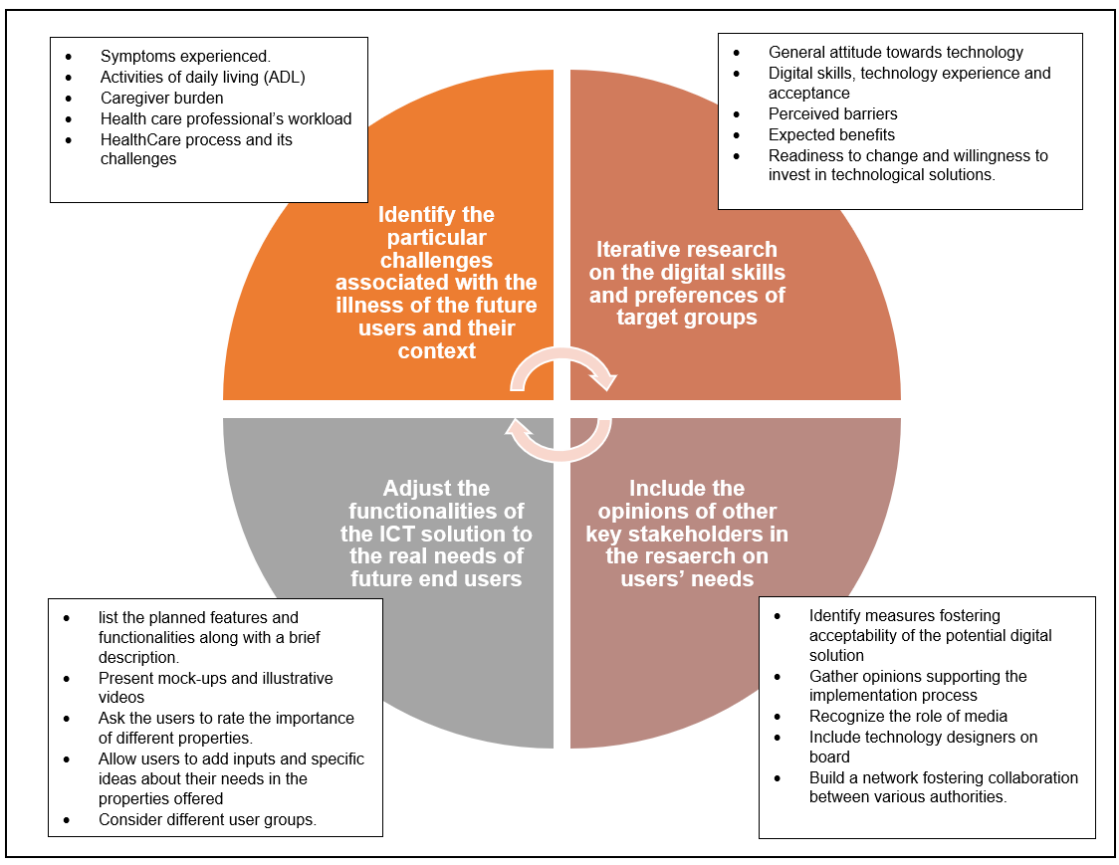
A model for research on users’ needs that covers 4 main aspects. The proposed topics to be investigated under each aspect are presented. The model is to be used by projects that aim to develop ICT-based health care platforms adopting a user-centered design approach and targeting older people with neurodegenerative diseases and other chronic conditions. ICT: information and communication technology.

There is evidence indicating that starting the development process with a comprehensive study on users’ needs can prevent or help better manage the uncertainty and skepticism of some target users toward the technology [54]. In this context, we propose recommendations for a comprehensive methodology to be adopted in studies on users’ needs with regard to ICT-based health care solutions for dementia and PD, and when the development process uses the UCD approach. These recommendations have been derived from the findings of this paper and other results of research on end-user needs reported elsewhere [33]. As there is an overlap between our findings and those of previous research [55], it is relevant to consider these recommendations when targeting older populations with other chronic diseases. The recommendations are grouped in a model that covers 4 aspects (Figure 1), which are explained in the following text.

#### Identify the Particular Challenges Associated With the Illnesses of Future Users and Their Context

One of the main aims when conducting research on users’ needs is to identify the specific challenges faced by potential users and how they are being tackled. Older people with chronic conditions, particularly those with NDDs, face everyday problems that also affect their relatives and families. There is a large heterogeneity of situations linked to the cognitive and mobility capabilities of future end users. Therefore, future studies should focus on actual functions and abilities rather than the diagnosis and medical symptoms and should assess the individual disease experience and evolution. This will provide a better understanding of the influence of concrete symptoms on everyday life abilities and mental abilities [56]. From our findings, looking only at the diagnosis could be misleading. For instance, 71% of our patients were diagnosed with PD, which is a motor disease. However, based on an in-depth analysis, both personalized memory support and reminders were mostly required as functionalities to be included in digital health care solutions.

Furthermore, dementia, PD, and comorbidities have implications for not only the people diagnosed with these conditions but also other end-user groups, namely, health care professionals and caregivers. Therefore, the needs and problems of these groups, such as caregiver burden and health care professionals’ workload, need to be considered in the ICT design and development processes.

#### Iterative Research on the Digital Skills and Preferences of Target Groups

Digital skills vary greatly across different end-user groups, and thus, there is no single solution that fits all groups. Therefore, when developing an ICT-based health care platform, it is important to consider the previous technology literacy and digital abilities of the intended end users, together with their social support to use the technology [57]. From PROCare4Life, we recommend investigating users’ technology experience and acceptance as an integral part of the research on users’ needs. Applying both objective and subjective measures, we argue that this approach could help ensure the inclusivity of the digital solution developed.

Another important aspect is identifying the barriers that might hinder the intended users’ adoption of the specific digital solution. Acknowledging those barriers at the early stage of the development might support the developers to accommodate the solution and provide alternatives when eventual barriers might be identified. For instance, in our research, most of the potential end users did not accept the use of in-depth cameras owing to privacy concerns. Identifying this issue, the project development team created a modular system that worked with and without cameras, depending on the end users’ preferences. Additionally, the technical team worked on updating the code with the in-depth cameras and the real-time software to avoid the storage of any patient’s images. Meanwhile, when developing a digital platform that includes various devices (smartphones, wearables, and cameras), we recommend exploring the acceptance of each one of the integrated devices among the intended target groups, which will help reduce the risk of the devices being left unused.

Furthermore, another recommendation is to consider exploring the participants’ opinions regarding the expected benefits and how much they are willing to invest in the technology being developed. Recognizing these facts as early as possible will support developers in aligning the design of the technology with the reported expectations, making it more relevant for intended users.

#### Adjust the Functionalities of the ICT Solution to the Real Needs of Future End Users

Similar to the findings of other studies, our research on users’ needs identified that personalization and ease of use are features that are often discussed in connection with technology [58-61]. The transformation of those needs and the realization of personalized technical features require proper research on the needs of end users. If this is not followed, there is a risk that the developed technology will only integrate the assumptions, limitations, and biases of the research and development teams. Questions, such as “What do elderly people living with chronic diseases need?” “How can technology contribute to those needs?” and “Which functionalities and features need to be integrated?”, must be researched and addressed considering the different target end-user groups.

We recommend conducting interactive discussions with future end users, providing explanations to them, and relating symptoms to functionalities as a way to support the verbalization of their needs. Initiatives, such as showcasing interface mock-ups and demonstrating various technical features using illustrative videos or screenshots of the design and functionalities throughout the development process, can foster collaborative design and co-creation of the digital solution. This, in turn, can enhance its future acceptance and usability through the incorporation of adjustments whenever challenges are encountered by the research team or end users. Our suggestions align with previous research [62,63] that reported the difficulties faced by most end users, particularly older patients, when imagining how the desired functionalities are to be integrated into digital solutions.

#### Include the Opinions of Other Key Stakeholders in Research on Users’ Needs

The UCD and co-design approaches are common in the development of technologies for older populations, with the first aiming to involve users in the development process and the second bringing together different stakeholders to collaborate on the design [64,65]. While both approaches have been recommended when developing ICT-based health care platforms [31,66], only few studies have reported the actual use of their principles [67]. In the PROCare4Life research, the importance of including other key stakeholders beyond end users has been recognized. In fact, the introduction of the voices of experts in areas linked to the future exploitation of ICT systems, such as scientific research, market, policy, and media, has not only enriched research on users’ needs and fostered the overall UCD approach, but also helped anticipate the future implementation of ICT-based platforms. Based on our findings, this dialog with experts provides valuable ideas that can be considered in efforts to develop a well-accepted digital solution and facilitate the implementation process in the medium to long term.

Considering the growing role that the media has played in recent years and the possible use of the media to shape the awareness of many people in digital health care, the importance of bringing media experts on board in future research needs to be emphasized. It is also important for technology designers to take part in such research studies as they have been referred to as fundamental stakeholders in digital health care solutions, and their needs and preferences should be identified and considered [68].

### Strengths and Limitations

This study was affected by the restrictions associated with COVID-19 and the contingency measures. For practical and safety reasons, most of the interviews were conducted online. Video calls were set up to reduce the distance between the interviewee and interviewer at times of social distancing due to the COVID-19 pandemic. Whenever possible, English was the language used. However, participants were free to decide on the use of their native language, as selective translation and transcription of the interviews were conducted later during the data analysis process. No language barrier was reported during the interviews. The flexibility to conduct both remote and in-person interviews, when necessary, demonstrates the study’s adaptability to changing circumstances. This aspect highlights the strength of the methodological approach.

The involvement of highly experienced experts from various disciplines and a variety of countries within the European Union has brought diverse knowledge, expertise, and perspectives to this study. The inclusion of professionals from diverse backgrounds, such as media, market, decision making, and academia, was essential for obtaining a comprehensive understanding of the elements impacting the acceptability and implementation of ICT-based health care platforms in the context of NDDs. The inclusion of the perspectives of media professionals was instrumental in shaping public perceptions, potentially contributing to increased acceptance and understanding of the platform’s benefits.

The diversity of experts and the quality of their experiences strengthened the validity and relevance of the findings obtained in the framework of this study, thereby solidifying the robustness of the obtained results. However, the lack of representation of technology experts or designers among interviewees was a main limitation of this study. Aiming to investigate the technical perspectives, our methodology relied on the experience brought by the different groups of key stakeholders included and the technology experts who were active in the PROCare4Life consortium. Nevertheless, future studies need to include the voices of technology designers and experts, and make sure that their needs and ideas are well represented in research on users’ needs.

The thematic analysis along with the deductive-inductive approach in this study used a framework derived from the question categories in the interviews rather than using other existing technology acceptance models (eg, Technology Acceptance Model [TAM] and Unified Theory of Acceptance and Use of Technology 2 [UTAUT-2]). This is mainly due to the fact that these models focus primarily on the users and their application requires the users to test or use the technology before providing their opinions [69-71]. Hence, considering the setting of our study, the use of a deductive-inductive approach yielded mostly consistent results. Furthermore, the methodological model suggested by this study could be a foundation for a framework to be adopted by future projects, ensuring that other key stakeholders beyond users are involved at an early stage of the development process of the platform.

The research on users’ needs was intended mainly to support and guide the development of the PROCare4Life platform, meaning that questions and discussions regarding the specific design of PROCare4Life were integrated into the study guidelines. This might be interpreted as a limitation for the generalization of our results and, in turn, the recommendations proposed. Nevertheless, the questions included in the study were mostly general, and they were analyzed in our results. Furthermore, we demonstrated the alignment of our results with the literature and even pointed out the importance of considering the specificity of every project in our recommendations.

### Future Directions

Gangas et al [72] recently published a paper highlighting the lessons learned from pilot 3 of PROCare4Life. The paper emphasized that developing multidisciplinary collaboration is effective for identifying and managing the challenges related to ICT platforms, both in the development and implementation phases. Aligning with our results, we argue that responding to the various needs of end users in ICT-based health care platforms requires a shared dialog between different experts, such as researchers, policy makers, technology developers, and media experts. It is possible to address the barriers to ICT adoption among older populations, such as technology complexity and digital literacy, by providing a tailored platform. Future projects are required to plan and budget for personalized training measures among end users, ensuring that the time needed for training on the technology is considered. Furthermore, overcoming the costs of technology adoption can be facilitated by offering tailored pricing models that accommodate the idiosyncrasies implicit in reimbursement systems unique to regions or countries. However, considering the importance of maintaining sustainable funding for digital health solutions, specific research needs to be conducted to identify the various existing funding opportunities and explore other ideas with different stakeholder groups.

The role of the media in shaping public awareness, including awareness among older populations, has been noted in this study. However, the precise impact of this role on the acceptance of ICT solutions among older people with chronic conditions needs to be further investigated. More studies should assess and report results, and there should be consideration of which media channels and strategies need to be used and their effectiveness, what type of content needs to be incorporated, and how can the messages be crafted and tailored for this target group.

### Conclusion

The data obtained from this study highlighted the importance of the UCD approach in ensuring the involvement of users in the development of ICT-based platforms and integrating their needs. The most common challenges that hinder the acceptability and implementation of ICT-based health care platforms can be addressed by creating synergies among the efforts of users, academic stakeholders, developers, policy makers, and decision makers. To allow future projects to base the development of ICT-based health care platforms on a comprehensive understanding of potential users, this study outlined the following recommendations that can be integrated when conducting research on users’ needs: (1) properly identify the particular challenges faced by future user groups without neglecting their social and clinical contexts; (2) iteratively assess the digital skills of future users and their acceptance of the proposed platform; (3) align the functionalities of the ICT platform with the real needs of future users; and (4) involve key stakeholders to guide the reflection on how to implement the platform in the future.

## Abbreviations

ICT: information and communication technology
NDD: neurodegenerative disease
PD: Parkinson disease
PROCare4Life: Personalized Integrated Care Promoting Quality of Life for Older People
UCD: user-centered design

## Data Availability

The data sets generated during or analyzed during this study are available from the co-author MM or the corresponding author upon reasonable request.
